# Exploratory study on autoantibodies to arginine-rich human peptides mimicking Epstein-Barr virus in women with post-COVID and myalgic encephalomyelitis/chronic fatigue syndrome

**DOI:** 10.3389/fimmu.2025.1650948

**Published:** 2025-09-19

**Authors:** Friederike Hoheisel, Kathrin Maria Fleischer, Kerstin Rubarth, Nuno Sepúlveda, Sandra Bauer, Frank Konietschke, Claudia Kedor Peters, Annika Elisa Stein, Kirsten Wittke, Martina Seifert, Judith Bellmann-Strobl, Josef Mautner, Uta Behrends, Carmen Scheibenbogen, Franziska Sotzny

**Affiliations:** ^1^ Institute of Medical Immunology, Charité – Universitätsmedizin Berlin, Corporate Member of Freie Universität Berlin and Humboldt Universität zu Berlin and Berlin Institute of Health, Berlin, Germany; ^2^ Institute of Virology, Helmholtz Munich, Munich, Germany; ^3^ Children’s Hospital, School of Medicine, Technical University of Munich, Munich, Germany; ^4^ Technical University of Munich, Munich, Germany; ^5^ Institute of Biometry and Clinical Epidemiology, Charité – Universitätsmedizin Berlin, Corporate Member of Freie Universität Berlin, Humboldt-Universität zu Berlin, Berlin, Germany; ^6^ Faculty of Mathematics & Information Science, Warsaw University of Technology, Warsaw, Poland; ^7^ CEAUL - Centro de Estatística e Aplicações da Universidade de Lisboa, Lisbon, Portugal; ^8^ Berlin Institute of Health at Charité – Universitätsmedizin Berlin, BIH Center for Regenerative Therapies (BCRT), Berlin, Germany; ^9^ DZHK (German Center for Cardiovascular Research), partner site, Berlin, Germany; ^10^ Experimental and Research Center (ECRC), Charité - Universitätsmedizin Berlin, Corporate Member of Freie Universität Berlin, Humboldt Universität zu Berlin and Berlin Institute of Health, Berlin, Germany; ^11^ NeuroCure Research Centre, Charité - Universitätsmedizin Berlin, Corporate Member of Freie Universität Berlin, Humboldt Universität zu Berlin and Berlin Institute of Health, Berlin, Germany; ^12^ Max Delbrück Center for Molecular Medicine in the Helmholtz Association (MDC), Berlin, Germany; ^13^ German Center for Infection Research (DZIF), Berlin, Germany

**Keywords:** autoantibodies, cross-reactivity, EBV, arginine-rich peptides, post-COVID syndrome, ME/CFS

## Abstract

**Introduction:**

Epstein-Barr virus (EBV) infection is a well-established trigger and risk factor for both myalgic encephalomyelitis/chronic fatigue syndrome (ME/CFS) and post-COVID syndrome (PCS). In previous studies, we identified elevated IgG responses to arginine-rich (poly-R) sequences within the EBV nuclear antigens EBNA4 and EBNA6 in post-infectious ME/CFS (piME/CFS). Building on these findings, this exploratory study examines IgG reactivity to poly-R-containing EBV-derived peptides and homologous human peptides in women with PCS and ME/CFS.

**Methods:**

IgG reactivity to poly-R containing peptides derived from EBNA4 and EBNA6, and homologous human 15-mer peptides and the corresponding full-length proteins, was assessed using a cytometric bead array (CBA) and a multiplex dot-blot assay. Serum samples were analyzed from 45 female PCS patients diagnosed according to WHO criteria, including 26 who also met the Canadian Consensus criteria for ME/CFS (pcME/CFS), 36 female patients with non-COVID post-infectious ME/CFS (piME/CFS), and 34 female healthy controls (HC).

**Results:**

Autoantibodies targeting poly-R peptide sequences of the neuronal antigen SRRM3, the ion channel SLC24A3, TGF-β signaling regulator TSPLY2, and the angiogenesis-related protein TSPYL5, as well as full-length α-adrenergic receptor (ADRA) proteins, were more frequently detected in patient groups. Several of these autoantibodies showed positive correlations with core symptoms, including autonomic dysfunction, fatigue, cognitive impairment, and pain.

**Conclusion:**

This exploratory study identify autoantibodies directed against EBV mimicking arginine-rich sequences in human proteins, suggesting a potential role for molecular mimicry in the pathogenesis of PCS and ME/CFS.

## 1 Introduction

Severe Acute Respiratory Syndrome Coronavirus 2 (SARS-CoV-2) infection has been shown to induce a range of autoantibodies (1–3). Several studies have demonstrated antinuclear autoantibodies (ANA), as well as autoantibodies directed against G protein-coupled receptors (GPCRs), and various neuronal, muscular, and other intra- and extracellular proteins in post-COVID syndrome (PCS). There is evidence of sequence similarities between autoantigenic targets and SARS-CoV-2 proteins (3). Autoantibodies have also been found to be associated with core symptoms of the disease (1, 3–7). Importantly, two recent studies have shown that transferring IgG from PCS patients can induce similar symptoms in mice (8, 9). In the study by Santos Guedes de Sa et al., patients showed a broad pattern of autoantibodies reactive to neuronal tissues and meninges. IgG from individual patients induced distinct symptoms such as pain, hypersensitivity, or loss of coordination in mice.

PCS shares many overlapping symptoms with myalgic encephalomyelitis/chronic fatigue syndrome (ME/CFS), which in most patients is also triggered by an infection, too. However, PCS encompasses a broader spectrum of symptoms and phenotypes, and only a smaller subgroup meets the diagnostic criteria for ME/CFS. ME/CFS is defined by the key symptoms of severe fatigue, and post-exertional malaise (PEM), a worsening of symptoms following even minor physical or mental activity (10, 11). Patients with ME/CFS typically experience greater physical impairment and more severe symptoms than most individuals with PCS.

There is also increasing evidence for the role of autoantibodies in post-infectious (pi) ME/CFS (12, 13). GPCR autoantibodies have been found to be elevated in a subgroup of ME/CFS and to correlate with symptom severity and alterations in magnetic resonance imaging (MRI) indices (14–17). Epstein-Barr virus (EBV) is a well-known trigger for ME/CFS, and EBV reactivation during COVID-19 is a risk factor for PCS (18–20). Altered antibody responses to EBV antigens have been identified in ME/CFS, with emerging evidence of cross-reactivity to human proteins. A recent study identified autoantibodies to myelin basic protein in ME/CFS, a known autoantigen in multiple sclerosis with sequence similarity to Epstein-Barr nuclear antigen (EBNA) 1 (21). In a previous study, we analyzed IgG reactivity against over 3000 overlapping 15-mer peptide sequences derived from 14 EBV proteins. Patients with ME/CFS exhibited significantly increased IgG binding to multiple EBV-derived peptides (22). Notably, several epitopes were identified within a repetitive region of the EBNA6 protein, which shares sequence homology with various human proteins. Elevated IgG reactivity was also observed against the full-length EBNA6 protein. Subsequent bioinformatic analysis revealed enriched IgG responses against arginine-rich (poly-R) motifs within EBNA6 and EBNA4 in patients with post-infectious ME/CFS patients. These poly-R sequences are of particular interest because they are common motifs in several human proteins involved in immune regulation and neural function, and such homology could promote cross-reactive antibody responses (23, 24). Further, poly-R motifs carry a high positive charge, and immune responses against these sequences can be broadly cross-reactive as demonstrated in a previous study (25).

Given the overlapping clinical features of PCS and ME/CFS, along with the known role of EBV as a trigger in a subset of ME/CFS patients and the involvement of EBV reactivation during COVID-19 as risk factor for PCS (18–20), we hypothesized that autoantibodies targeting EBV-derived poly-R motifs and their homologous human sequences would be detected more frequently in both conditions. We further hypothesized that elevated autoantibody levels indicate functional relevance and therefore would correlate with core clinical symptoms.

In the present study, we investigated IgG reactivity to poly-R–containing peptides from the EBV proteins EBNA4 and EBNA6, their homologous human sequences, and the corresponding full-length proteins in patients with PCS and ME/CFS. We further assessed the relationship between antibody levels and core clinical symptoms.

## 2 Materials and methods

### 2.1 Study participants

For this exploratory study, female participants aged 18 to 59 years were recruited from the ME/CFS clinic of the Charité Fatigue Centre, Berlin. Serum samples were collected from 45 PCS patients suffering from moderate to severe fatigue, with 26 of them fulfilling the Canadian Consensus Criteria (CCC) for the diagnosis of ME/CFS (pcME/CFS). In the case of 2 of 26 pcME/CFS patients and 3 of 19 PCS patients with a disease duration of less than 6 months, the diagnosis was confirmed at month 6. In addition, we included serum samples from 36 other post-infectious ME/CFS (piME/CFS) patients. These patients developed ME/CFS after an infection most commonly following non-SARS-CoV-2 viral respiratory tract infections or EBV infection. Moreover, we included 34 female HCs, of whom 26 reported a SARS-CoV-2 infection at least 6 months prior. Information about a previous SARS-CoV-2 infection were not available for the piME/CFS study group.

Detailed cohort information is displayed in **Table 1** (**Table 1**, **Supplementary Data 1**). Disease and symptom severity were assessed in patients with appropriate questionnaires. The functional disability was evaluated using the Bell score, ranging from 0 to 100 (with 100 for no restrictions) (26). Physical function and daily activities were assessed using the Short Form Health Survey 36 (SF-36), ranging from 0 to 100 (greatest to no restrictions) (27). PEM severity was evaluated according to the brief DSQ-PEM questionnaire and scores ranging from 0 to 46 calculated (no to frequent/severe PEM) (28). The severity of the key symptoms fatigue, pain, and cognitive impairment was quantified using a Likert scale (1 = no symptoms to 10 = severe symptoms). Autonomic dysfunction was assessed using the Composite Autonomic Symptom Score 31 (COMPASS-31), ranging from 0 to 100 (no to strongest impairment) (29).

**Table 1:** Cohort characteristics.

	*HC*	*piME/CFS*	*pcME/CFS*	*PCS*	*p-value*
Number of participants*: *Total (n); peptide IgG (n_I_) and protein IgG study (n_II_)*	n=34; n_I_=30; n_II_=25	n=36; n_I_=32; n_II_=32	n=26; n_I_=25; n_II_=22	n=19; n_I_=19; n_II_=16	n. a.
***Age [years]***	37 (22-56)	40 (20-59)	41.5 (26-58)	39 (18-54)	0.356
***time since SARS-CoV-2 infection [months]***	10.4^#^ (5.2-28.5) no prev. inf.: n=7	n. a.^$^	9.6 (4.5-24.0)	7.3 (5.1-27.0)	0.074
***Disease duration [years]***	n. a.	3.1 (0.50-24)	0.75 (0.33-1.92)	0.58 (0.42-2.25)	**<0.0001**
***Bell***	n. a.^$^	30 (20-60)	30 (20-50)	50 (30-80)	**0.0003**
***SF-36 PF***	n. a.^$^	35 ^####^ (0-70)	32.5 (0-65)	60 (10-90)	**0.0076**
***Fatigue Score***	n. a.^$^	8.3 ^##^ (6.3-10.0)	8.5 ^#^ (6.0-10.0)	7.3 ^#^ (4.8-9.8)	**0.0023**
***Cognitive Score***	n. a.^$^	7.2 (1.7-9.0)	7.0 (2.0-9.3)	7.0 ^#^ (2.3-9.0)	0.564
***Muscle Pain***	n. a.^$^	7.0 ^#^ (1.0-10.0)	6.5 (1.0-10.0)	6.0 (1.0-10.0)	0.500
***Headache***	n. a.^$^	6 (1-10)	7 (1-10)	6 ^#^ (1-9)	0.462
***PEM***	n. a.^$^	35.5 ^######^ (18.0-46.0)	39.5 ^##^ (25.0-46.0)	31.0 ^###^ (15.0-41.0)	**0.0002**
***COMPASS-31***	n. a.^$^	43.9 ^#^ (21.5-69.6)	38.9 ^#^ (12.1-65.7)	39.8 ^#^ (2.0-56.3)	0.1991

Continuous variables are presented as median and range. Not all serum samples from each study group were analyzed in both assays (*). The total number of participants (n) as well as the number of samples included in the peptide IgG (n_I_) and protein IgG assays (n_II_), respectively, are indicated for each study group. No information about a previous SARS-CoV-2 infection are available in the piME/CFS group and disease/symptom questionnaire scores are not available for the HC group (*^$^)*. Univariate comparisons of study groups were done using the Kruskal–Wallis test. A two-tailed p-value of <0.05 was considered statistically significant [indicated in bold]. Information was unavailable from one ( *^#^) up to* six ( *^######^ ) patients of the respective study group.* [n. a., not assessed; prev. inf., previous infection].

Routine laboratory parameters were determined at the Charité diagnostics laboratory Labor Berlin GmbH (Berlin, Germany). Study data, including clinical and routine laboratory parameters, were collected and managed using REDCap electronic data capture tools hosted at Charité Universitätsmedizin Berlin (30, 31).

This study was approved by the Ethics Committee of Charité Universitätsmedizin Berlin (EA2/067/20, EA2/066/20) and is per the 1964 Declaration of Helsinki and its later amendments. The participants provided written informed consent.

### 2.2 Analysis of IgG reactivity to EBV and human peptides by peptide CBA

#### 2.2.1 Identification of human candidate peptide sequences

In a previous study using a regression model for binary outcomes a significantly elevated IgG reactivity was identified against two arginine-rich (poly-R) EBV peptides in a subset of ME/CFS patients compared to HC, namely EBNA4_0529 and EBNA6_0070 (23). To study the potential role of the candidate epitopes in eliciting cross-reactive antibodies against human antigens, we searched for sequence alignments between the EBNA4_0529 and EBNA6_0070 antigen sequences and human proteins. This search was conducted in the web server Protein Blast from the NCBI (https://blast.ncbi.nlm.nih.gov/Blast.cgi). Database searching was restricted to the human sequences (taxid:9606) of the RefSeq_select (https://www.ncbi.nlm.nih.gov/refseq/refseq_select/). The default search parameters of the Blast algorithm were automatically adjusted for short input sequences when searching for candidate sequence alignments. We considered sequence alignments when the query coverage was greater than 50% and the identity was at least 75% and identified 82 alignments with human protein sequences for EBNA6_66/70 and 35 for EBNA4_0529. Among all sequences identified we selected eight sequences of human proteins whose dysfunction could be of potential relevance to the core pathomechanisms currently recognized in PCS and ME/CFS. This selection was based on peer-reviewed literature linking these proteins to autonomic and neuronal dysfunction, mitochondrial dysfunction and vascular dysregulation, thereby ensuring that the selection process was systematic rather than arbitrary. In the case of membrane proteins, we checked for the localization of the sequences, and only sequences outside the membrane domains were included in this study.

#### 2.2.2 Cytometric bead array

Serum IgG binding to EBV EBNA epitopes and their respective human mimicry sequences was analyzed using a peptide cytometric bead assay (CBA). For this, BD functional CBA beads were first conjugated with streptavidin (SAV, Roche, Cat. no. 11721674001) using Sulfo-SMCC technology according to the manufacturer’s protocol (BD™ Cytometric Bead Array Functional Bead Conjugation Buffer Set, Cat. no. 558556). In brief, the disulfide bridges on functional beads were reduced with Dithiothreitol within one hour incubation (Applichem, Cat. no. A1101,005), and in parallel, SAV was modified by reacting one hour with Sulfo-SMCC (ThermoFisher Scientific, Cat. No. 22322). Following re-buffering using a Bio-Spin^®^ 30 Tris Column (Bio-Rad, Cat. No. 7326231), the modified SAV was combined with the functional beads for another hour. Finally, the SAV-coated beads were stabilized by a 15 minute treatment with N-ethylmaleimide (ThermoFisher Scientific, Cat. no. 23030). After SAV binding was confirmed using a PE-coupled anti-SAV antibody (BioLegend^®^, Cat. no. 410504) by flow cytometry, the biotinylated 15-mer peptides were coupled to individual SAV-conjugated bead populations. Briefly, the SAV-conjugated beads were incubated with the corresponding biotinylated 15-mer peptides in a final concentration of 0.1 µM in Capture Bead Diluent (BD™ Cytometric Bead Array Human Soluble Protein Master Buffer Kit, Cat. no. 558265) for one hour. Sequences of the EBV, the human mimicry, and the non-sense EBV EBNA1 scrambled (negative control) peptides are shown in **Table 2**. The EBNA6_740 peptide was included as a positive control, as more than 90% of ME/CFS and HC exhibit high antibody levels against it (22). Biotinylated peptides were synthesized via Fmoc-based solid phase peptide synthesis (SPPS) on polystyrene resin and purified by RP-HPLC by JPT Peptide Technologies GmbH (Berlin, Germany). A unique bead labeling of APC and APC-Cy7 allowed multiplexing of up to 15 peptide-coupled bead populations. The measurement of patient and control serum IgG was performed according to the manufacturer’s protocol (BD™ Cytometric Bead Array Human Soluble Protein Master Buffer Kit, Cat. no. 558265). In detail, the sera were incubated for one hour in a final dilution of 1:100 with the prepared beads. After washing, Fcγ fragment specific R-Phycoerythrin AffiniPure™ Goat Anti-Human IgG (Jackson Immuno Research, Cat. no. 109-115-098) in a final dilution of 1:50 was added for additional two hours. Samples were measured on a CytoFlex LX flow cytometer, data were acquired with CytExpert 2.4, and analyzed using FlowJo 10.8.2. Each bead set included a bead population coated with a scrambled sequence of EBV EBNA1 (EBNA1_530scr) to control for unspecific IgG binding. The median fluorescence intensity (MFI) of detected unspecific binding was subtracted from each MFI of detected bound serum IgGs to peptide-coupled beads of interest (background correction). Corrected MFIs ≤ 0 means no detectable binding of IgG to the peptides and were set to 0. Since we found an intra-assay variability of up to 30%, a positive reactivity was defined as MFI > 30% compared to the MFI signal of IgG binding to the scrambled negative peptide (EBNA1_530scr) of the respective serum.

**Table 2:** 15-mer peptides of EBV and human sequences with homologous sequences beeing underlined. The number after “_” in the peptide ID refers to the starting position of peptide in the EBV or human reference protein sequence.

	Peptide ID	Amino acid sequence	Protein
EBV peptides	EBNA6_66	NRGWMQRIRRRRRRR	Epstein-Barr nuclear antigen 6
	EBNA6_70	MQRIRRRRRRRAALS	Epstein-Barr nuclear antigen 6
	EBNA4_529	PQQPRAGRRGPCVFT	Epstein-Barr nuclear antigen 4
	EBNA6_740	QPAPQAPYQGYQEPP	Epstein-Barr nuclear antigen 6
	EBNA1_530scr	FRSGRTPLLPCQMAL	Scrambled sequence of Epstein-Barr nuclear antigen 1
Human EBNA6-66/70 homologous peptides	ADRA1B_369	RGRRRRRRRRRRLGG	α-1B adrenergic receptor
	ADRA1D_422	RRRRRRRPLWRVYGH	α-1D adrenergic receptor
	ADRA2C_289	SAAAERRRRRGALRR	α-2C adrenergic receptor
	SRRM3_38	AGRRRRRRRRRRRSR	Serine/arginine repetitive matrix protein 3
	SLC24A3_8	DRARRRRRRRRRRDL	Sodium/potassium/calcium exchanger 3 NCKX3
	PLD6_29	WLRSRRRRPRREALF	Phospholipase D family member 6; Mitochondrial cardiolipin hydrolase
	TSPYL2_185	SSRRRRRRRRRKQRK	Testis-specific Y-encoded-like protein 2
Human EBNA4-529 homologous peptide	TSPYL5_133	KNAPRVGNRRGPAGK	Testis-specific Y-encoded-like protein 5

### 2.3 Analysis of IgG reactivity to EBV and human full-length proteins

To detect serum IgG reactivity to EBV proteins and potential human autoantigens, sera were tested in a multiplex dot blot assay as described (32). Briefly, 5 µl of concentration-adjusted recombinant His_6_-tagged proteins were spotted on a nitrocellulose membrane. Following a blocking step with 5% milk powder in PBS the membrane was co-incubated overnight at 4°C with an anti-His_6_ antibody (clone 3D5) and 1:1000 diluted serum in a 3% milk buffer. Following washing, membranes were incubated for 1.5 hours with fluorescence-labeled anti-mouse IgG antibody (LI-COR^®^ IRDye 680) and anti-human IgG antibody (LI-COR^®^ IRDye 800) to quantify the amount of spotted proteins and binding of human IgG. The membranes were scanned in a LI-COR^®^ Odyssey FC scanner that reports results as arbitrary fluorescence units (AFU), returning a CW700 and a CW800 reading for each dot on the membrane corresponding to the protein concentration (anti-His_6_) and the human serum reactivity, respectively. A standard curve of recombinant His_6_-tagged human IgG, as well as solvent (8 M urea) and Ni-NTA agarose-affinity enriched mock-transfected HEK293T cell lysate, were used for specific standardization and background correction, enabling blot-to-blot comparability.

Autofluorescence signals caused by the nitrocellulose membrane or the solvent as well as any possible fluorescence due to serum reactivity against HEK293T proteins were subtracted from readings for antigenic proteins. Background-subtracted AFU values for proteins in the CW800 and CW700 channels were converted to normalized arbitrary values using a simple linear regression model drawn from values obtained for serial dilutions of recombinant IgG for each individual membrane. Next, the quotient of normalized CW800 and CW700 values was formed to compensate for potential differences in the amount of sample protein spotted on the membrane. This normalized AFU value describes sera IgG binding against EBV and human proteins. Normalized AFUs ≤ 0 means no detectable binding of IgG to the target protein and were set to 0. All normalized AFU values >0 were considered as positive IgG reactivity.

### 2.4 Statistical analysis

Statistical data analyses were performed using R Version 4.2.1 and GraphPad Prism Version 9.5.1. All scripts for the analyses conducted in R are openly accessible via GitHub at https://github.com/KerstinRubarth/IMMME_SP4, and raw data is accessible as **Supplementary Data 1**.

Patient characteristics are presented as median and range for continuous variables stratified by study group. Univariate comparisons of independent groups were done using the Kruskal–Wallis test. Two-tailed tests were used with a significance level of 5%.

For each study group specific IgG binding was descriptively analyzed, presenting the medians and Interquartile Ranges (IQRs) of the untransformed, background-corrected values (peptide IgG binding), or untransformed, normalized values (protein IgG binding), respectively. Due to the skewed distributions of the IgG levels, data were visualized using a log_10_ transformation, which provided the most effective scaling for the plots. Univariate pairwise comparisons of each patient group with the HC group were performed with the untransformed values using the nonparametric Mann-Whitney test. Two-tailed tests were used with a significance level of 5%.

In addition, IgG binding was analyzed using linear models, with the group as the independent variable and IgG binding as the dependent variable. To account for skewed distributions and outliers in IgG levels, a log-transformation with a base of 2 was applied. Negative values and zeros were set to 0.01 for this analysis. Subsequently, linear models were fitted using the R-function ‘lmFit’ from the R-package ‘limma’, and model parameters were estimated utilizing the empirical Bayes method (R-function ‘eBayes’). The results were presented through volcano plots with a log2 fold change (FC) cutoff at one and a p-value cut-off of 0.1.

The frequency of positive IgG reactivity was expressed as a percentage. Pairwise comparisons of categorical variables between patient groups with HC group, respectively, was done using two-tailed Chi-Squared test with a significance level of 5%.

Correlations of IgG binding and clinical scores were analyzed for each group separately by presenting heatmaps displaying Spearman-type correlation coefficients ≥ ± 0.3, indicating at least small to moderate correlations (33). Spearman correlation was used due to the non-normal distribution of the data. P-values less than 0.05 were considered as statistically significant and were indicated by * in the heatmaps. Due to the exploratory nature of the study, no correction for multiple testing was applied. Instead, the focus was on investigating effect sizes, such as correlations and log-fold changes. p-values are provided for orientation and should be interpreted in a hypothesis-generating rather than a confirmatory manner.

## 3 Results

### 3.1 IgG reactivity against EBV and homologous human peptides

In a previous study employing a regression model for binary outcomes, a significantly elevated IgG reactivity was observed against two arginine-rich EBV-derived EBNA peptides in piME/CFS patients compared to HC (23). Using NCBI´s Protein Blast and the RefSeq_select databases, we identified human proteins with high sequence homology to arginine-rich EBNA6_70/66 peptides. These proteins selected for their potential pathophysiological relevance to ME/CFS include α-adrenergic receptors (ADRA1B, ADRA1D, ADRA2C), the sodium/potassium/calcium exchanger 3 (SLC24A3), the nuclear serine/arginine repetitive matrix protein 3 (SRRM3), the mitochondrial phospholipase D family member 6 (PLD6) and the intracellular testis-specific Y-encoded-like protein 2 (TSPYL2) (**Table 2**). All aligned sequences share a characteristic poly-R motif. In the α-adrenergic receptors, this motif was localized in the cytoplasmic C-terminus; in SLC24A3, it was found in the cytoplasmic N-terminus. In PLD6, a mitochondrial outer membrane protein, the poly-R sequence was likewise located within the cytoplasmic region. For EBNA4_529, sequence homology was identified with the nuclear testis-specific Y-encoded-like protein 5 (TSPYL5), which contains three arginines (**Table 2**).

**Figure 1:**
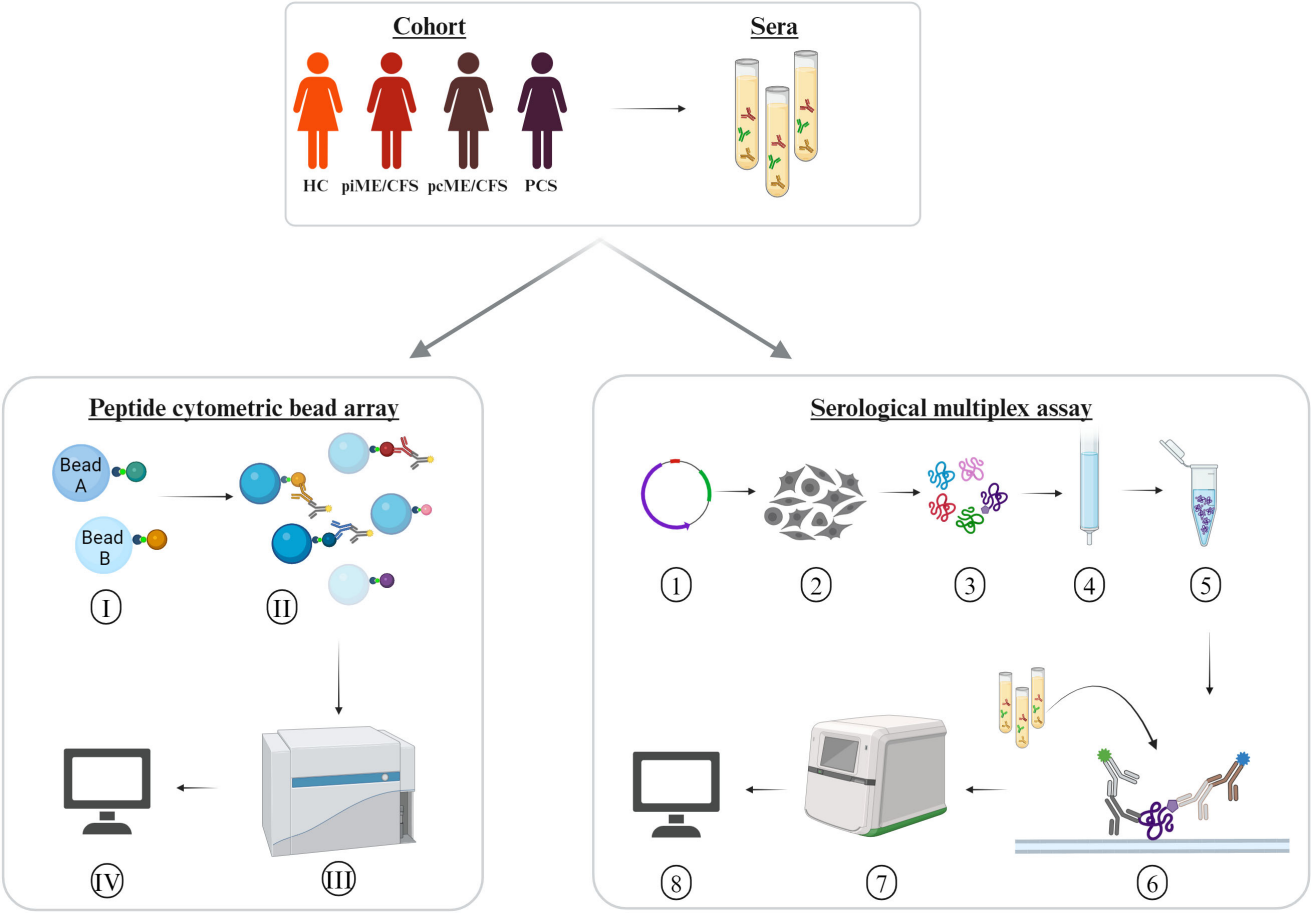
Experimental design. Sera of age-matched female patients with myalgic encephalomyelitis/chronic fatigue syndrome (ME/CFS) after SARS-CoV-2 (pcME/CFS), or after other infectious triggers (piME/CFS), as well as post-COVID syndrome (PCS) were analyzed in comparison to healthy controls (HC). Peptide cytometric bead array (CBA) was performed for detection of IgG reactivity to peptides of interest. (I) Coupling of the beads with 15-mer peptides of interest. (II) Binding of the serum IgG reactive against the peptides of interest, and (III) measurement of the serum IgG reactivity in a multiplex approach unsing CytoFlex LX device. (IV) Data analysis of median fluorescence intensity (MFI). Serological multiplex array measurement of serum IgG reactivity to proteins of interest. (1) Expression vectors for C-terminally His6-tagged proteins of interest were generated using a pcDNA3.1-derived plasmid. (2) Transfection into HEK293T cells. (3) Harvesting and lysing cells after four days. (4) Purification of His6-tagged proteins over to Ni2+-NTA columns. (5) Verification of protein integrity and identity. (6) Addition of the proteins to a nitrocellulose membrane and incubation with an anti His6-tag monoclonal mouse antibody. (7) Detection of bound human and mouse antibodies with secondary antibodies in a near-infrared detection system. (8) Antibody reactivity to proteins were quantified by normalization of arbitrary fluorescence units (AFU) relative to the amount of spotted protein and a standard.

In the present study, we analyzed antibody reactivity to these EBV EBNA peptides and their homologous human counterparts, as well as to the corresponding full-length proteins, in female PCS, pcME/CFS, piME/CFS patients, and HC. The cohorts were age-matched (**Table 1**). Time since last SARS-CoV-2 infection was comparable between the post-COVID patient groups and the HC group; 26 of HCs reported a previous SARS-CoV-2 infection, with a median of 10.4 months before study participation. However, patients with piME/CFS had a significantly longer disease duration (median 3.1 years) than pcME/CFS (median: 0.75 years) and PCS (median: 0.58 years) patients. ME/CFS patients exhibited greater physical impairment, more severe fatigue, and more pronounced PEM than PCS patients. The EBNA6_740 peptide was included as a positive control, as our previous study had shown that most patients and HCs displayed high antibody levels against this peptide (22). An overview of the study design is provided in **Figure 1**.

**Figure 2:**
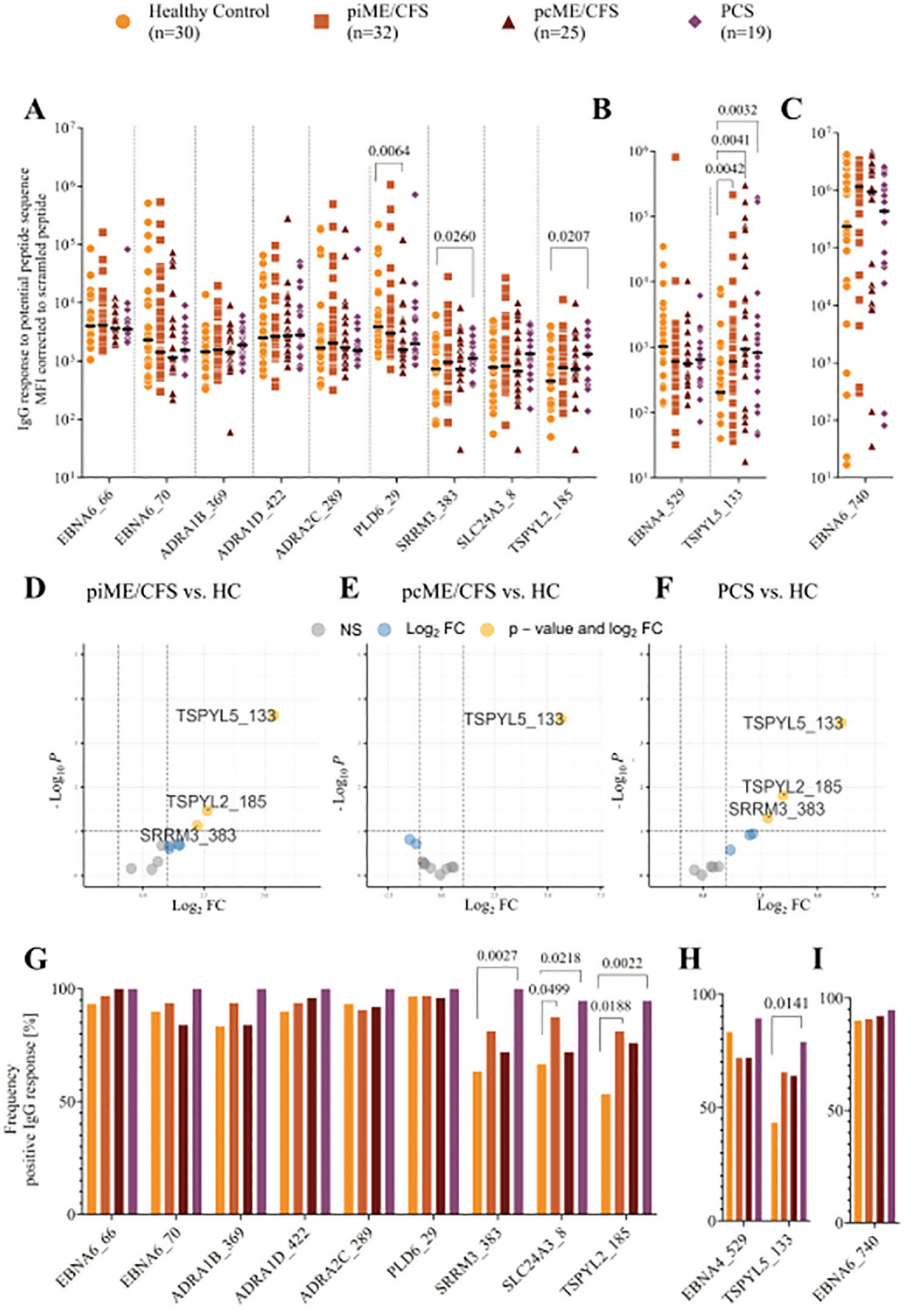
Analysis of IgG reactivity to peptides by CBA. Shown are the individual serum IgG reactivities as Mean Fluorescence Intensity (MFI) corrected to the MFI of IgG binding to scrambled EBV EBNA1 peptide (EBNA1_530scr) visualized using Log_10_ transformation **(A-F)** and the frequency of positive IgG reactivity in % **(G, H)** of the whole patient cohorts (piME/CFS, pcME/CFS, PCS) and the healthy controls (HC). The IgG reactivities against **(A, G)** EBNA6_66/70 and homologous human peptides, **(B, H)** EBNA4_529 and homologous human peptide TSPYL5_133, as well as **(C, I)** EBNA6_740 were determined. Statistical pairwise comparison of the level of specific IgG binding between all patient cohorts and the HC cohort was done with the untransformed, background-corrected values using the Mann-Whitney-U test **(A-C)**. For the comparisons of IgG frequencies between study groups Chi-Squared test was used **(G-I)**. P-values less than 0.05 of two-tailed tests were considered statistically significant. The Limma analysis **(D-F)** plotted for 12 variables and filtered for p-value <0.1 (negative values and zeros were set to 0.01 for this analysis). The enhanced volcano plots compare the different patient cohorts piME/CFS **(D)**, pcME/CFS **(E)** and PCS **(F)**, each to healthy controls (HC).

We detected IgG reactivity against EBNA6 and EBNA4 peptides, as well as against all the homologous human sequence peptides, in the sera of both healthy controls and patients (**Figure 2**). In PCS patients, IgG binding was significantly increased for the EBNA6_66/70 homologous sequences TSPYL2_185 and SRRM3_383 (**Figure 2A**). Additionally, all patient groups showed significantly elevated levels of IgG against the EBNA4_529 homolog TSPYL5_133 compared to HCs (**Figure 2B**). Linear model analysis using the Limma framework confirmed significantly higher IgG reactivity to SRRM3_383, TSPYL2_185, and TSPYL5_133 in piME/CFS and PCS patients, and to TSPYL5_133 in the pcME/CFS cohort relative to HCs (**Figures 2D-F**).

Analysis of the frequency of IgG-positive individuals within each cohort revealed antibodies against the EBNA6 poly-R sequences, as well as the ADRA and PLD6_23 peptides, in nearly all PCS patients, and in most ME/CFS and HC individuals (**Figure 2G**). IgG reactivity to the SLC24A3_8, TSPYL2_185, and SRRM3_383 peptides was observed in 40 – 70% of the HCs but in 90 – 100% of PCS; for TSPYL2_185 and SLC24A3_8 peptides, reactivity was also significantly more frequent in piME/CFS patients than in HCs (**Figure 2G**). IgG reactivity to EBNA4_529 was found in approximately 75% of all samples, but more patients showed reactivity to the homologous peptide TSPYL5_133 (**Figure 2H**). As observed in our previous study, strong IgG responses to EBNA6_740 were present in 80 – 95% of all subjects (**Figures 2C, I**).

**Figure 3:**
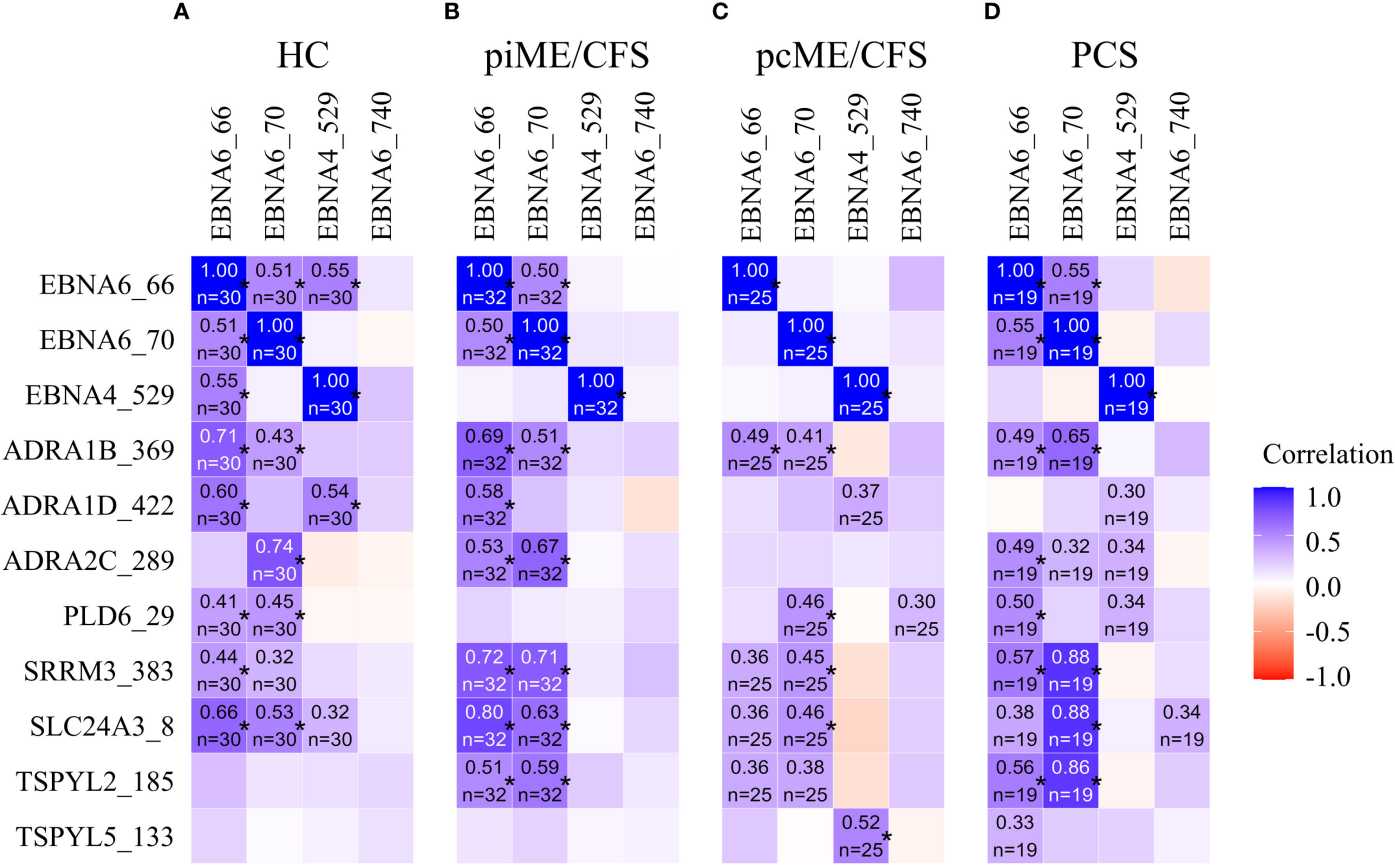
Heatmaps presenting the Spearman`s correlation coefficient r ≥ 0.3 of the correlation between IgG reactivity to EBV EBNA peptides and homologous human peptides in HC **(A)**, piME/CFS **(B)**, pcME/CFS **(C)** and PCS **(D)**, respectively. (*p-values < 0.05).

We further explored the potential cross-reactivity of IgG to EBV-derived and homologous human poly-R peptide sequences using Spearman`s correlation analysis (**Figures 3A-D**). Positive correlations between IgG levels targeting EBNA6_66/70 IgG and most homologous human peptides were observed across all cohorts. A correlation of IgG reactivity against EBNA4_529 and TSPYL5_133 was exclusively found in pcME/CFS. No correlations were observed for reactivity with the non-homologous EBN6_740.

### 3.2 IgG reactivity against EBNA and human full-length protein

Next, we studied the IgG reactivity to full-length EBNA proteins and the human proteins using a multiplex assay. Since no validated cutoff for positive antibody reactivity against these proteins exists to date, we present background-corrected, normalized arbitrary fluorescence units (AFU) for each protein, as described in the method section.

**Figure 4:**
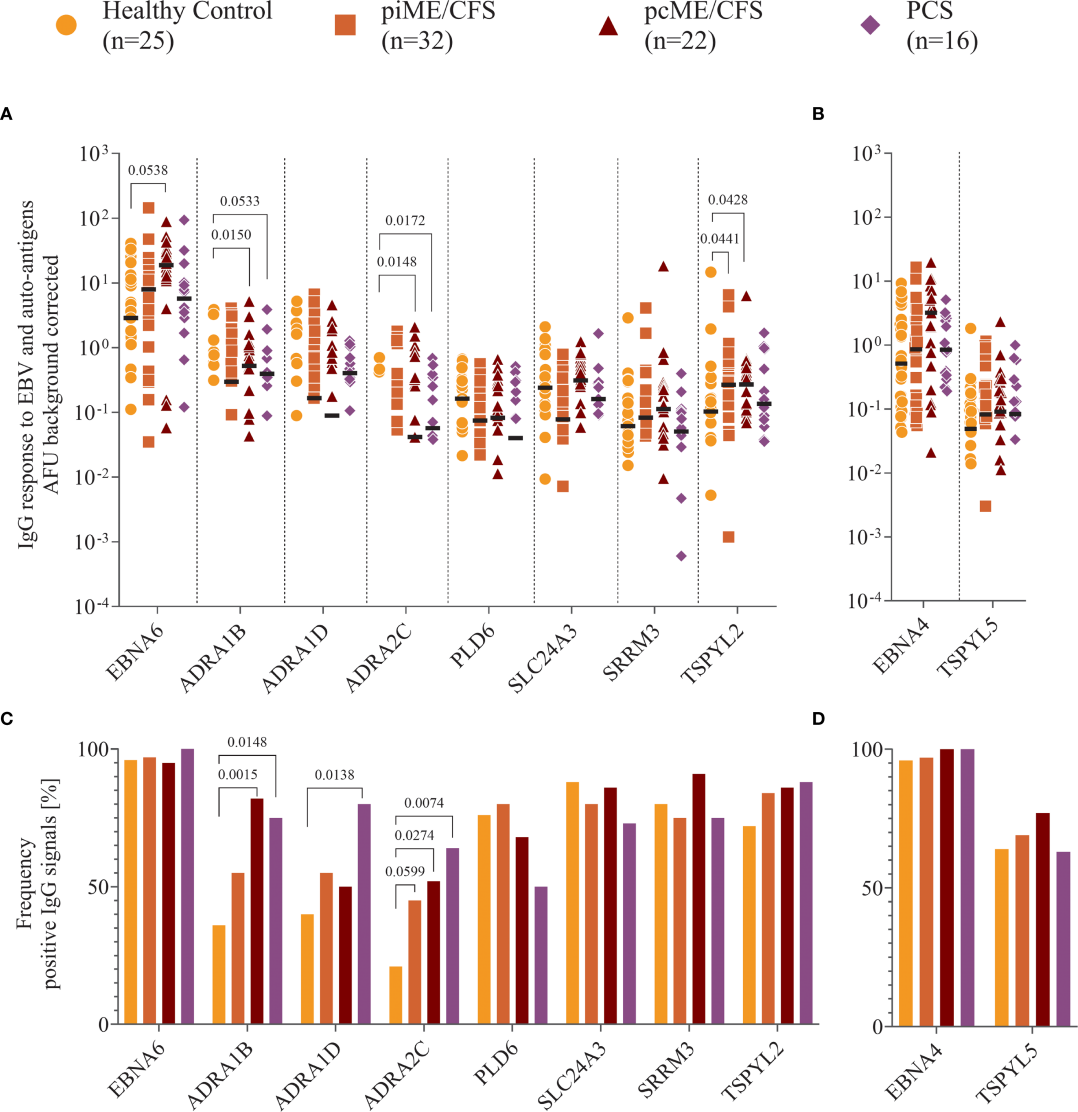
Analysis of IgG reactivity to proteins. Shown are **(A, B)** the individual serum IgG reactivity in normalized arbitrary Unit (AFU) visualized using Log_10_ transformation and **(C, D)** the frequency of positive IgG reactivity in % of the whole patient cohorts (piME/CFS, pcME/CFS, PCS) and the healthy controls (HC). The IgG reactivity against **(A, C)** EBNA6 and human proteins, **(B, D)** EBNA4 and human TSPYL5 proteins were determined. Statistical pairwise comparison of the level of specific IgG binding between each patient group and the HC group was done using the Mann-Whitney-U test **(A, B)**. For the comparisons of IgG frequencies between study groups Chi-Squared test was used **(C, D)**. A two-tailed p-value of <0.05 was considered statistically significant.

We observed IgG binding to EBNA6 and EBNA4 proteins in most samples, with higher reactivity to EBNA6 in patient cohorts compared to HCs, reaching statistical significance in the pcME/CFS group (**Figure 4A**). IgG responses to the human proteins were detectable in all study cohorts, although signals were generally low in many individuals. Antibodies targeting ADRA proteins were present in 20 – 40% of the HCs, but were significantly more frequent (**Figure 4C**) and showed higher titers in PCS and ME/CFS patients (**Figure 4A**). While the frequency of IgG reactive against TSPYL2 was similar across groups (**Figure 4C**), both ME/CFS cohorts exhibited higher autoantibodies levels compared to HCs (**Figure 4A**). Comparable levels and frequencies of IgG binding to EBNA4 and its homolog TSPYL5 were found across all study groups (**Figures 4B, D**).

### 3.3 Association between IgG reactivity and symptom severity

**Figure 5:**
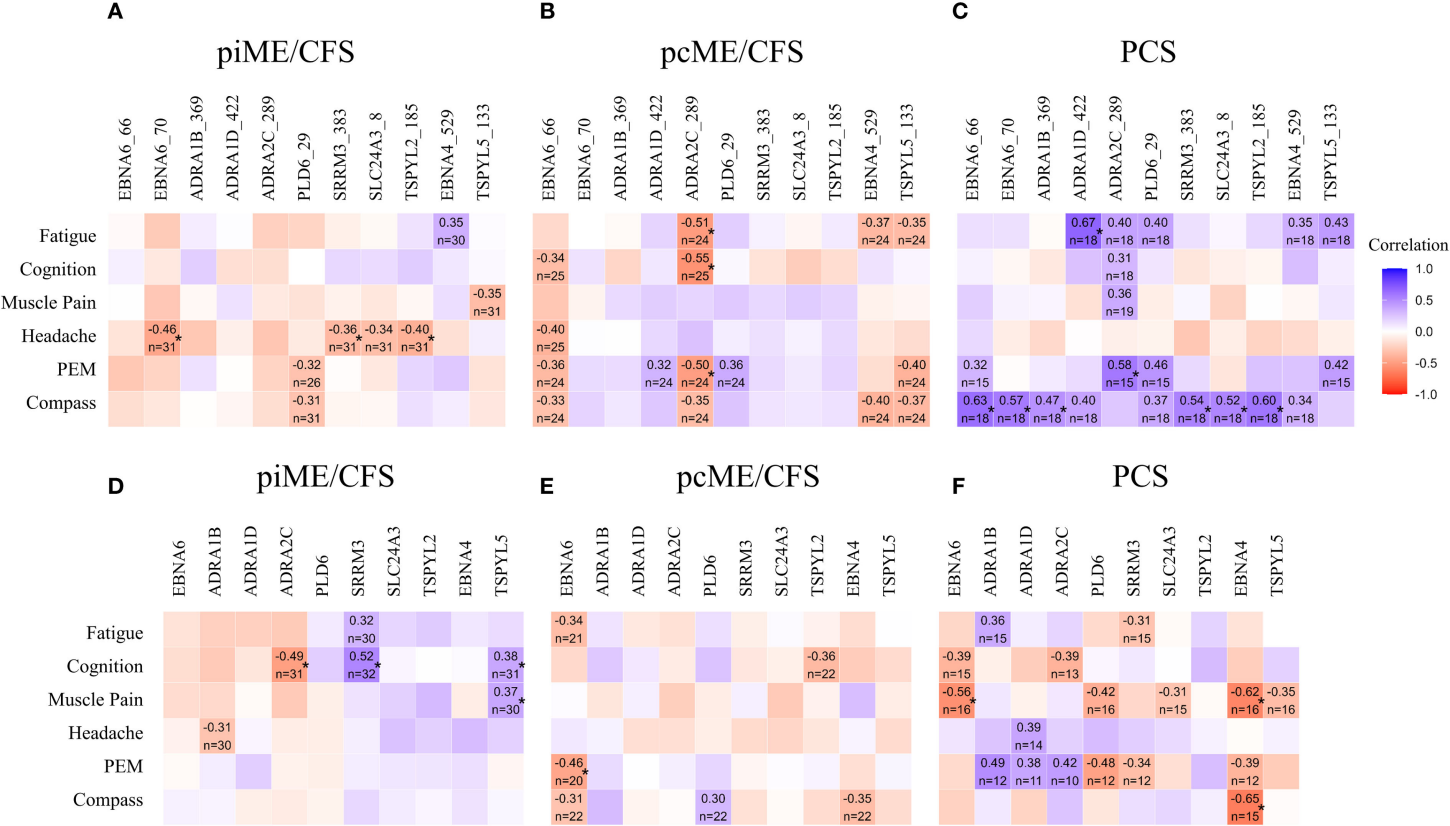
Associations of autoantibodies with symptom severity. IgG reactive to EBV and human peptides **(A-C)** and to corresponding proteins **(D–F)** were analyzed by Spearman correlation. Heatmaps presenting the Spearman correlation coefficient r ≥ 0.3 (*p-values < 0.05).

We next examined whether autoantibody levels were associated with the severity of key symptoms using Spearman correlation analyses (**Figure 5**). In PCS patients, significant positive correlations were observed between most antibody levels, including those to EBNA6_66/70, and autonomic dysfunction assessed by COMPASS score. IgG responses to two ADRA peptides also showed significant associations with fatigue and PEM, while several other correlations showed positive trends (**Figure 5C**). Although no significant correlations between antibodies to the full-length proteins and symptom severity were found in PCS, there was a noteworthy trend toward higher IgG levels to all three ADRA proteins being associated with PEM severity (**Figure 5F**).

In the ME/CFS cohorts, no significant positive correlations were observed between autoantibodies to peptides and symptom severity (**Figures 5A, B**). However, in the piME/CFS group, significant positive correlations were found between autoantibodies to SRRM3 protein and cognitive impairment, and between antibodies to TSPYL5 protein and both cognition and muscle pain (**Figure 5D**). In contrast to PCS, the pcME/CFS group exhibited several inverse correlations between IgG reactivity to ADRA2C_422 peptides and symptoms, such as fatigue, cognitive impairment, and PEM (**Figure 5B**). Similar associations were also found when symptom severity was compared between patients with high versus low autoantibody levels (**Supplementary Data 2**).

## 4 Discussion

**Figure 6:**
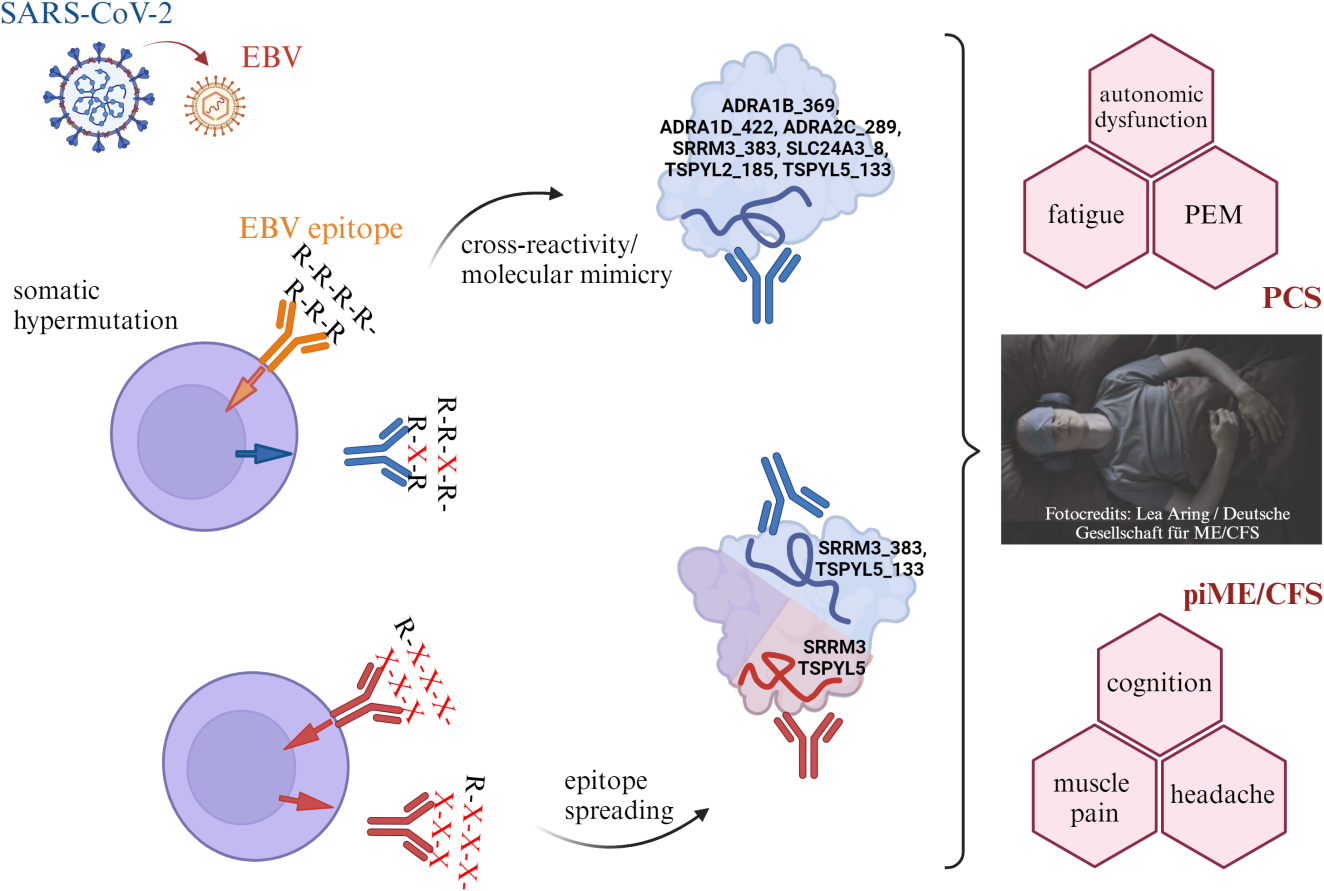
Proposal for a conceptual framework to explain the findings of this study. EBV reactivation which may be triggered by COVID-19 may activate B cells, and induce the production of antibodies targeting arginine-rich sequences within EBNA4 and EBNA6 as well as homologous sequences in human proteins. Through somatic hypermutation, antibody specificity can shift, potentially enhancing cross-reactivity with poly-R motifs in host proteins. This antibody reactivity may lead to sustained immune activation and further stimulation of B cells, resulting in the production of additional autoantibodies directed against other epitopes of the same self-antigens. Binding of such autoantibodies to their targets may interfere with the physiological functions of the affected proteins, potentially contributing to autonomic, mitochondrial, or vascular dysfunction—and ultimately to the development of clinical symptoms. Created with BioRender. https://BioRender.com/m23y013.

This study characterized antibody reactivity to human peptides with high sequence homology to arginine-rich (poly-R) sequences of EBV, as well as to corresponding human proteins, in patients with PCS and ME/CFS. The proteins identified here have known functions potentially relevant to central ME/CFS and PCS pathophysiology, including roles in autonomic regulation (ADRA), neuronal signaling (SRRM3), mitochondrial function and TGF-β signaling (TSPYL2), vascular tone and calcium signaling (SLC2A43), and endothelial integrity and angiogenesis (TSPYL5). The detection of some poly-R-reactive antibodies in healthy controls is not unexpected, as low-level natural antibodies are a normal component of the immune repertoire and may contribute to immune hemostasis. In disease states, however, such antibodies may occur at higher titers, exhibit altered affinity, or target functionally critical epitopes, thereby becoming potentially pathogenic. Elevated levels of several autoantibodies in PCS and ME/CFS compared to healthy controls, along with positive correlations to the severity of key clinical symptoms, underscore their potential clinical relevance. The observed correlations between IgG reactivity to EBNA6_66/70 and homologous poly-R containing human peptides suggest the possibility of cross-reactivity or molecular mimicry. **Figure 6** presents a conceptual framework that integrates our findings. The associations between levels of several autoantibodies and symptom severity imply that they may interfere with the function of the target proteins involved in autonomic regulation, mitochondrial activity, and vascular function.

We observed distinct differences in autoantibody titers and their correlation with symptoms across the three patient cohorts. PCS patients showed the highest levels and frequencies of autoantibodies to several human peptides. In line with this, autoantibodies to several poly-R peptides, including ADRA, were significantly associated with the severity of autonomic dysfunction, fatigue, and PEM in PCS. More frequent autoantibodies to the ADRA protein were found in all patient cohorts. Given the central role of adrenergic receptors in autonomic function, this finding aligns with previous studies that have linked such autoantibodies to symptom severity in PCS and ME/CFS (7, 34). It is tempting to speculate that the inverse correlations between IgG reactivity to ADRA2C_422 peptide and symptoms found in pcME/CFS, but not in PCS, in this study may indicate epitope spreading (**Figure 6**).

Higher IgG reactivity to both TSPYL2 peptide and its full-length protein was observed across all patient cohorts. TSPYL2 is known to regulate TGF-β signaling and can inhibit Sirtuin-1 (SIRT1) activity, a mechanism that could result in mitochondrial dysfunction (35, 36). Elevated TGF-β levels have been reported in ME/CFS patients in several studies (37).

Further elevated IgG reactivity to SLC24A3, SRRM3, and TSPYL5 peptides was also observed in PCS and piME/CFS cohorts. SLC24A3 is highly expressed in brain, skeletal, and smooth muscles and is known to regulate intracellular calcium levels and arterial smooth muscle contractility (38). A single nucleotide variant in SLC24A3 has been identified as a risk factor for migraine, a common comorbidity in PCS and ME/CFS (39). SRRM3 and TSPYL5 are likewise expressed in the brain (40, 41). No increased IgG reactivity to the corresponding full-length proteins of these three antigens was detected in patients compared with healthy controls. However, it may be possible that in patients these autoantibodies exhibit altered affinity or recognize other functionally critical epitopes as a result of epitope spreading. Support for a potential relevance of antibodies against SRRM3 and TSPYL5 proteins comes from the observed association between their levels and cognitive impairment, and between TSPYL5 and muscle pain, in piME/CFS patients. These protein-directed autoantibody responses may have been initially triggered by IgG reactive to the poly-R peptides. Since no direct correlation between IgG reactivity to corresponding peptides with symptoms was observed in piME/CFS, epitope spreading remains a plausible underlying mechanism (**Figure 6**).

The observed correlations between autoantibody levels with symptom severity gain further relevance when considering the known function of these proteins. SRRM3, which is expressed in the brain, is a regulator of alternative splicing essential for motor coordination and contributes to the switch of gamma-aminobutyric acid (GABA)ergic signaling from excitatory to inhibitory (40, 42). Enhanced sensitivity to light and sound in ME/CFS is hypothesized to result from low GABAergic activity. Notably, a single nucleotide variant in SRRM3 has been associated with ME/CFS (43). In multiple sclerosis, autoantibodies to SRRM3 have been detected years before the disease onset (44). TSPYL5 is known to be an angiogenic regulator with a key role in maintaining endothelial integrity, functionality, and proliferation (41). Several studies have demonstrated cerebral and muscular hypoperfusion as well as impaired microcirculation in ME/CFS (45–47). Recently, we observed that serum from PCS patients, but not from ME/CFS patients, promotes endothelial tube formation *in vitro* (48). This raises the possibility that the observed association of TSPYL5 autoantibodies with cognitive impairment, headache, and muscle pain in piME/CFS may be due to a potentially inhibitory effect on angiogenesis. The absence of such correlations in pcME/CFS remains unexplained but may relate to the significantly shorter disease duration in this group compared to piME/CFS patients (median 0.75 versus 3.1 years). It is plausible that the disease duration influences the composition of the autoantibody repertoire.

Notably, all self-antigens investigated in this study are expressed in the brain, and autoantibodies targeting neuronal tissue have been reported in PCS in several studies (1, 9). Although several poly-R reactive autoantibodies were associated with symptom severity in both PCS and ME/CFS, functional studies are needed to validate their pathogenic relevance. These include *in vitro* experiments with sequence-specific autoreactive IgGs or animal models immunized with EBNA6_66/70 or homologous peptides.

Poly-R motifs are found in numerous other viruses, including the Torque Teno virus (TTV), adenoviruses, and human papillomavirus HPV (25). In addition to EBV, adenoviruses and HPV are known as triggers of ME/CFS, and elevated TTV levels were recently described in ME/CFS (49). T cell clones reactive to the poly-R sequence of TTV were isolated from cerebrospinal fluid of a patient with multiple sclerosis (25). These T cell clones also reacted to poly-R motifs in several human autoantigens, including ADRA1B/D and ADRA2C, as well as the dopamine D2 receptor, purinergic P2X2 receptor, mitochondrial translocase TIM17, Toll-like receptor 9, and phosphatidylcholine transfer protein. These molecules may be relevant to the pathophysiology of ME/CFS.

These findings support the hypothesis that infections or reactivation of viruses containing arginine-rich motifs may contribute to the autoantibody findings in our study. We propose that such arginine-rich motifs, due to their widespread occurrence, may trigger autoreactive B and T cell responses through mechanisms such as cross-reactivity, molecular mimicry, or epitope spreading (**Figure 6**). However, binding studies are needed to confirm whether the identified poly-R reactive IgGs indeed cross-react with native host proteins.

It is important to acknowledge certain limitations of our exploratory hypothesis-generating study, including the restriction to females and the relatively small cohort sizes. Our preliminary findings should therefore be interpreted with caution and require validation in a larger, prospective, and confirmatory study. Additional validation cohorts will be essential to further evaluate the diagnostic potential of these autoantibodies and enable longitudinal analyses. Further, although the initial search was conducted across the complete human proteome, we subsequently prioritized proteins for autoantibody analysis based on their potential relevance to core pathomechanisms proposed for PCS and ME/CFS. This selection may have introduced bias and could have excluded other potentially relevant proteins.

In conclusion, we provide evidence that IgG autoantibodies directed against peptides containing poly-R motifs and their corresponding proteins are more frequent in PCS and ME/CFS patients than in healthy controls. Furthermore, we observed several associations between autoantibody levels and symptom severity, suggesting potential functional relevance. Cross-reactivity with conserved viral motifs may contribute to the heterogeneity of autoantibody profiles and the clinical manifestations in PCS and ME/CFS. Our findings warrant further investigation into their pathogenic role, their diagnostic utility, and the development of drugs targeting autoantibodies.

## Data Availability

The raw data supporting the conclusions of this article will be made available by the authors, without undue reservation.
