# Supplementary material for: Exploratory study on autoantibodies to arginine-rich human peptides mimicking Epstein-Barr virus in women with post-COVID and myalgic encephalomyelitis/chronic fatigue syndrome
Source: Front Immunol. 2025 Sep 19;16:1650948. doi: 10.3389/fimmu.2025.1650948 (PMC12491002; doi:10.3389/fimmu.2025.1650948)
Supplement: Supplementary file 2 [file DataSheet2.pdf]

Supplemental data

Methods

Statistical analysis

In addition to correlation analysis, we conducted Mann-Whitney test to assess differences in the clinical parameters between two groups. The specific IgG responses were categorized into two groups: "High" and "Low", based on the median value of the data. If the median was found to be 0, all values equal to or less than 0 were classified as "Low", while values greater than 0 were categorized as "High".

Results

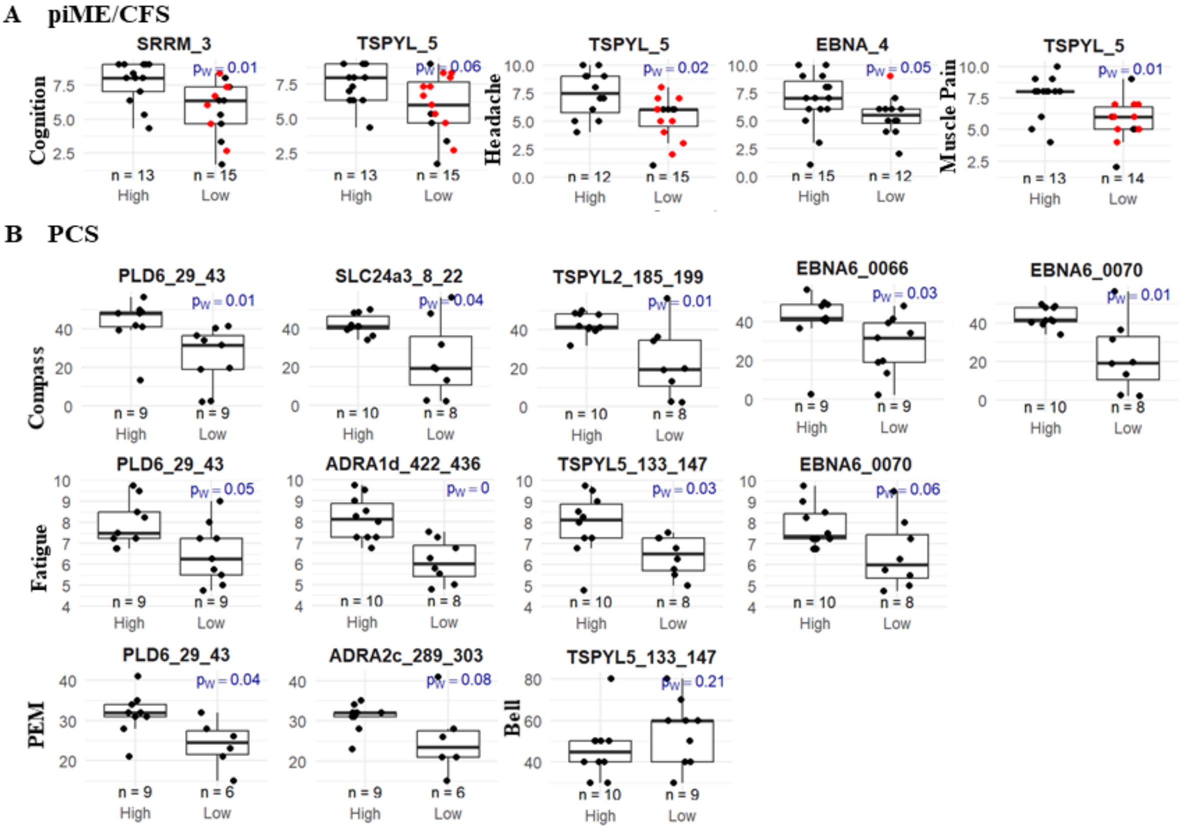

Supplementary Figure: Associations of autoantibodies with symptom severity. Shown are the clinical scores of patients with piME/CFS **(A)** or PCS **(A)** as median with interquartile ranges (IQR) comparatively for patient group with high or low IgG responses to human peptides or to corresponding proteins. Statistical comparison was done using the Mann-Whitney-U test. A two-tailed p-value of <0.05 was considered statistically significant. Samples without specific IgG binding were indicated as red dots.
